# Recent Progress on the Air‐Stable Battery Materials for Solid‐State Lithium Metal Batteries

**DOI:** 10.1002/advs.202307726

**Published:** 2023-12-10

**Authors:** Bingbing Cheng, Zi‐Jian Zheng, Xianze Yin

**Affiliations:** ^1^ College of Materials Science and Engineering, State Key Laboratory of New Textile Materials & Advanced Processing Technology Wuhan Textile University Wuhan 430073 China; ^2^ Ministry of Education Key Laboratory for the Green Preparation and Application of Functional Materials, Hubei Key Laboratory of Polymer Materials Hubei University Wuhan 430062 China

**Keywords:** air stability, battery material, lithium metal battery, safety, solid‐state battery

## Abstract

Solid‐state lithium metal batteries (SSLMBs) offer numerous advantages in terms of safety and theoretical specific energy density. However, their main components namely lithium metal anode, solid‐state electrolyte, and cathode, show chemical instability when exposed to humid air, which results in low capacities and poor cycling stability. Recent studies have shown that bioinspired hydrophobic materials with low specific surface energies can protect battery components from corrosion caused by humid air. Air‐stable inorganic materials that densely cover the surface of battery components can also provide protection, which improves the storage stability of the battery components, broadens their processing conditions, and ultimately decreases their processing costs while enhancing their safety. In this review, the mechanism behind the surface structural degradation of battery components and the resulting consequences are discussed. Subsequently, recent strategies are reviewed to address this issue from the perspectives of lithium metal anodes, solid‐state electrolytes, and cathodes. Finally, a brief conclusion is provided on the current strategies and fabrication suggestions for future safe air‐stable SSLMBs.

## Graphical abstract

Solid‐state lithium metal batteries (SSLMBs) have shown great potential in energy density and safety. The intrinsic defects arising from the chemical instability of the main components of battery when exposed to moist air, however, have restricted their practical application. In this review, the causes, consequences, and corresponding solution strategies of battery performance degradation are summarized when the battery components are exposed to humid air.

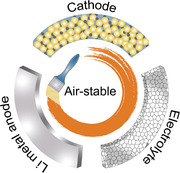

## 1 Introduction

Rechargeable lithium metal batteries (LMBs) are promising future energy storage devices due to their high output energies.^[^ ^1^, ^2^, ^3^, ^4^ ^]^ Among various candidates, solid‐state lithium metal batteries are particularly attractive because replacing liquid electrolytes with solid‐state electrolytes (SSEs) increases the energy density and safety of batteries.^[^ ^5^, ^6^, ^7^, ^8^ ^]^ However, despite their advantages, SSLMBs are still unsuitable for practical applications due to the chemical instability of their main components in humid air.^[^ ^9^, ^10^, ^11^ ^]^

The Li metal anode is an essential component of SSLMBs, but it is highly sensitive to air and moisture.^[^ ^12^ ^]^ When exposed to humid air, Li metal rapidly corrodes and becomes covered in a thick insulating layer consisting of LiOH, Li_3_N, and Li_2_CO_3_.^[^ ^13^, ^14^ ^]^ Furthermore, the reaction between Li and H_2_O produces flammable H_2_ gas and significant heat, which pose safety hazards.^[^ ^15^ ^]^ Consequently, Li metal must be processed in argon‐filled glove boxes. As the core components of SSLMBs, oxide‐based SSEs, sulfide‐based SSEs, and halide‐based SSEs have been developed as ion conductors due to their wide electrochemical stability windows and high ion conductivities.^[^ ^16^, ^17^, ^18^ ^]^ However, they are inherently sensitive to H_2_O and CO_2_.^[^ ^19^, ^20^ ^]^ For instance, garnet‐based SSEs can react with moisture in the air, which generates lithiophobic species that contaminate the electrolyte and result in poor contact between the electrolyte and electrodes, ultimately leading to large voltage polarization.^[^ ^21^, ^22^ ^]^ Similarly, sulfide‐based SSEs rapidly hydrolyze when exposed to air, releasing toxic H_2_S gas and deteriorating the cathodes.^[^ ^23^, ^24^ ^]^ Hydrophilic halide‐based SSEs readily transform into hydrates and other components, resulting in a rapid degradation of their ionic conductivity.^[^ ^25^ ^]^ To achieve a high specific energy, cathodes in SSLMBs often use lithium‐rich transition metal oxide materials.^[^ ^26^, ^27^, ^28^ ^]^ However, these high‐nickel cathodes can be exposed to air during the synthesis and electrode processing, causing the formation of inactive lithium‐containing compounds (such as Li_2_O, LiOH, Li_2_CO_3_, and LiHCO_3_) on the particle surfaces.^[^ ^29^, ^30^ ^]^ This leads to structural degradation and performance fading of the cathodes.

Therefore, enhancing the air insensitivity of battery materials is important for reducing battery processing costs and enhancing battery safety.^[^ ^31^ ^]^ Introducing artificial protective layers and doping elements at interfaces are two main strategies for enhancing the air/water stability of battery materials.^[^ ^32^, ^33^, ^34^, ^35^ ^]^ Artificial protective layers have attracted attention due to their diversity and long‐term protection, including hydrophobic layers and chemically‐inert layers. The inspiration design for hydrophobic layers comes from nature, as many naturally occurring surfaces show specific wettability.^[^ ^36^, ^37^, ^38^ ^]^ An example of this is lotus leaves, which demonstrate superhydrophobicity due to the synergistic effect of the wax layer and surface texture, which enables water droplets to move freely on their surface.^[^ ^39^, ^40^ ^]^ The low surface energy of the epicuticular wax endows the lotus leaves with their hydrophobicity. The numerous micro‐nanostructures present on the surface of lotus leaves can accommodate a significant amount of air within their spaces, leading to a transition from hydrophobic to superhydrophobic. In addition to the bioinspired hydrophobic protective layers, an air‐stable inorganic layer can also be used to protect battery components from air corrosion during electrode fabrication.^[^ ^41^, ^42^ ^]^ Overall, decorating the surface of battery materials with artificial protective layers can protect battery components from atmospheric corrosion to enable the construction of high‐performance SSLMBs.

**Figure 1:**
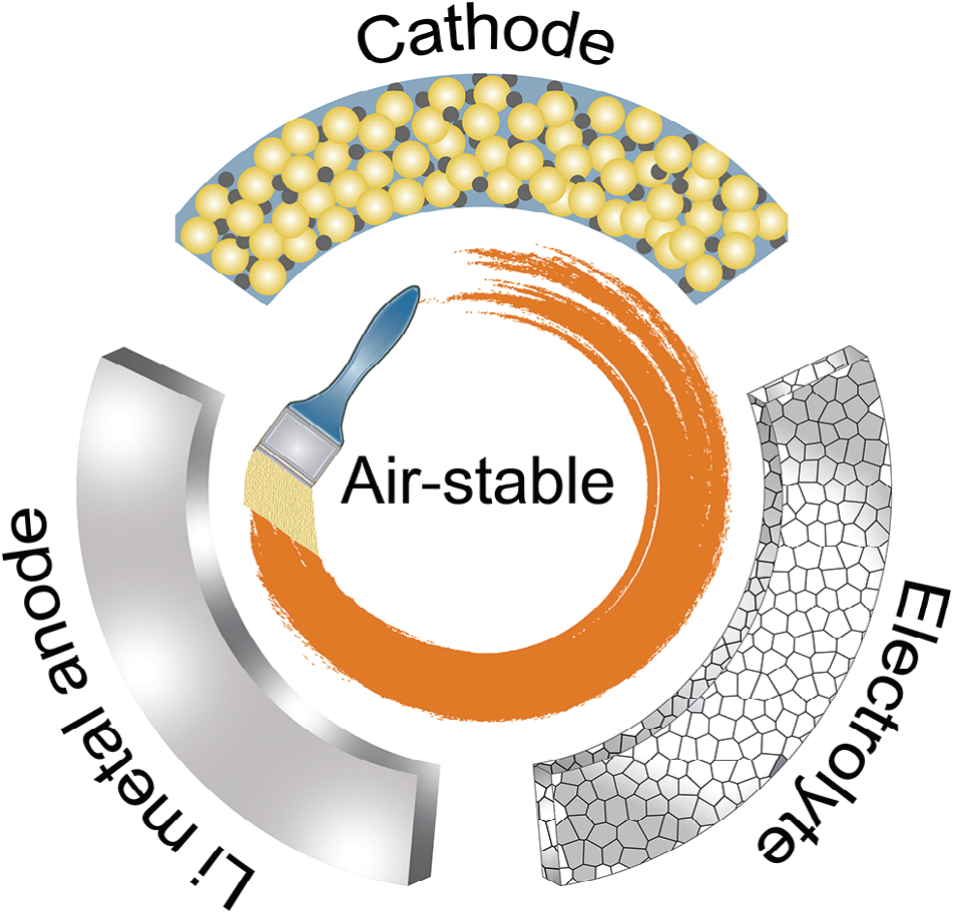
Schematic illustration of the design of air‐stable protective layers toward battery components for solid‐state lithium metal batteries.

In this review, we primarily focus on recent progress in the development of air‐stable SSLMBs to provide a systematic understanding of the causes and consequences of battery performance degradation when battery components are exposed to the air. Additionally, we review recent strategies for improving air stability, including advancements in Li metal anodes, solid‐state electrolytes, and cathodes (**Figure** **1**). Finally, we discuss challenges and opportunities associated with current strategies and propose future directions for the development of safe and air‐stable SSLMBs.

## 2 Air‐Stable Mechanism

During the preparation or storage of battery materials, their structure is inevitably compromised by moisture, oxygen, and carbon dioxide from the air, which may reduce the performance of the resulting battery. Transforming their surfaces from hydrophilic to hydrophobic by adopting a protective layer can protect them from attack by humid air. Thus, numerous efforts have been devoted to developing hydrophobic interfacial layers.

Inspired by lotus leaves, artificial super‐hydrophobicity layers have been designed to protect the surface of battery materials.^[^ ^43^ ^]^ The wetting behavior of a surface can be characterized by the water contact angle (WCA). On a smooth surface, the apparent WCA in the air can be explained by Young's equation,^[^ ^44^ ^]^

(1) $$\cos\;\theta_{0}=\left(\gamma_{\mathrm{sg}}-\gamma_{\mathrm{sl}}\right)/\gamma_{\mathrm{lg}}$$

where *θ*_0_ signifies the water contact angle on a solid surface, *γ*_sg_ represents the solid‐gas interfacial tension, *γ*_sl_ corresponds to the solid‐liquid interfacial tension, and *γ*_lg_ represents the liquid‐gas interfacial tension. A hydrophobic surface is defined by a WCA larger than 90°. According to Young's equation, decreasing the surface energy of the substrate is a prerequisite for fabricating hydrophobic surfaces.^[^ ^45^, ^46^ ^]^

In addition to surface energy, surface roughness is another decisive factor in regulating surface wettability. The Wenzel model was introduced to describe the influence of surface roughness on contact angle measurements and states that the contact angle of a liquid droplet on a solid surface is affected by microscopic depressions on the surface.^[^ ^47^ ^]^ In this model, a modified version of Young's equation can be used, which incorporates the effects of surface roughness. This modified equation is as follows:

(2) $$\cos\;\theta_{w}=\;r\cos\theta_{0}$$

where, *θ*_w_ denotes the wetting contact angle on a rough surface, and *r* is a roughness factor that is correlated with the surface area:

(3) $$r\;=\;\left(\mathrm{actual}\;\mathrm{surface}\right)/\left(\mathrm{geometric}\;\mathrm{surface}\right)$$

The variable *r* represents the ratio of the actual surface area to its apparent counterpart, and this value is always greater than 1. Equation (3) suggests that a higher surface roughness increases the degree of surface wettability, i.e., a hydrophobic surface will become superhydrophilic if *r* is increased. The surface free energy and surface roughness are the two key factors in designing hydrophobic surfaces. The surface free energy determines surface hydrophilicity or hydrophobicity, while surface roughness enhances these properties.^[^ ^48^, ^49^ ^]^

In addition to hydrophobic protective layers, other protective layers formed by chemically‐inert materials can also be adopted to protect battery materials from air corrosion. These well‐designed layers provide protection by densely packing on the surface of battery materials to reduce their air‐exposed area.

## 3 Air‐Stable Strategies

### 3.1 Li Metal Anode

As crucial components of high‐energy‐density rechargeable batteries, Li metal anodes have a high theoretical specific capacity of 3860 mA h g^−1^ and low reduction potential (−3.040 V vs standard hydrogen electrode).^[^ ^50^, ^51^, ^52^, ^53^ ^]^ However, the practical applications of Li metal anode are hindered by their high chemical reactivity. First, Li metal easily react with H_2_O, N_2_, O_2_, and CO_2_, leading to high requirements for anode processing and cell assembly. During storage and transportation, uneven and ion‐insulating surface layers arising from the unavoidable parasitic reactions between Li and air that cover on Li surfaceaggravate Li dendrite growth and deteriorate the electrochemical performance. Thus, there is an urgent need to fabricate air‐stable Li metal anodes for the industrial‐up production of Li metal batteries.

Surface engineering plays an important role in tuning the air stability of Li metal anodes.^[^ ^54^ ^]^ Fabricating a hydrophobic layer on Li anodes prevents the Li metal from being corroded by wet air and can also suppress Li dendrite growth and alleviate Li/electrolyte interfacial side reactions. Inspired by lotus leaves, surface components with low surface energies and micro/nanoscale hierarchical structures are critical for fabricating hydrophobic surfaces. In this context, many materials, including inorganic materials, organic materials, and organic‐inorganic hybrid materials, are designed to decorate the surface of Li metal anodes, thus improving their air stability.

#### 3.1.1 Inorganic Artificial Layers

**Figure 2:**
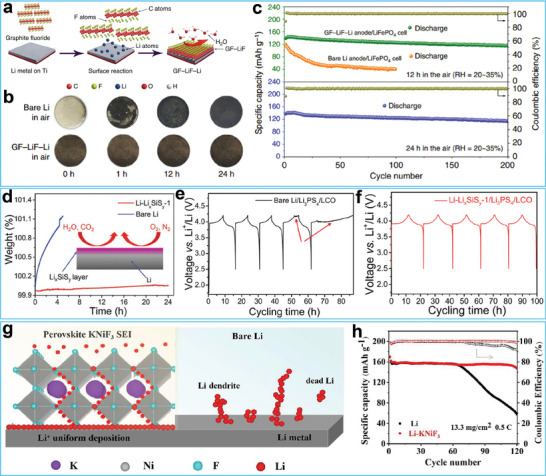
a) Schematic illustrating of the preparation process of GF (graphite fluoride)–LiF–Li anode. b) Air‐stable testing of bare Li metal anode and GF–LiF–Li anode. Reproduced with permission. c) Cycling performance of the anode after exposing in air.^[^ ^58^ ^]^ Copyright 2019, Springer Nature. d) Thermal gravimetric analysis of the bare Li anode and Li‐Li_x_SiS_y_ anode. e) Charge–discharge voltage profiles of Li/Li_3_PS_4_/LCO cell. f) Charge–discharge voltage profiles of Li‐Li_x_SiS_y_/Li_3_PS_4_/LCO cell. Reproduced with permission.^[^ ^63^ ^]^ Copyright 2019, Wiley‐VCH. g) Schematic illustration of Li^+^ deposition behavior on the surface of Li‐KNiF_3_ anode and bare Li anode. h) Cycling performance of the full cell based on bare Li metal anode and Li‐KNiF_3_ anode. Reproduced with permission.^[^ ^66^ ^]^ Copyright 2022, Elsevier.

Carbon materials, metallic materials, and other air‐stable inorganic materials can be adopted as protective layers to functionalize the Li metal anode, thus reducing its air sensitivity. Carbon materials are particularly favored due to their low density and high chemical inertness.^[^ ^55^, ^56^, ^57^ ^]^ In a recent study, Goodenough and co‐workers fabricated a hydrophobic interphase on a Li metal anode by incorporating graphite fluoride (GF) into molten Li.^[^ ^58^ ^]^ By utilizing the in situ reaction between GF and molten Li, as well as the low density of GF, a hybrid GF–LiF protective layer was constructed, as displayed in **Figure** **2**a. This layer protected the Li metal anodes by preventing reactions between Li metal anodes and humid air. Optical photographs confirmed that the GF–LiF–Li anode maintained its color, shape, and texture, even after exposure to humid air for 24 h (Figure 2b). Full cells based on the air‐treated GF–LiF–Li anode showed better charge‐discharge cycling performance than cells assembled with bare Li metal exposed to the air (Figure 2c).

Surface alloying is another straightforward and efficient technique for passivating the surface of Li metal anodes.^[^ ^59^, ^60^, ^61^, ^62^ ^]^ Li readily reacts with various metals to form alloys. Compared with pristine Li metal anodes, Li metal anodes covered with an alloy layer presented outstanding air stability. These alloy layers possessed a high affinity for Li ions, facilitated rapid ion diffusion, and exhibited excellent corrosion resistance, and mechanical strength. These characteristics enabled the protected Li metal anodes to undergo uniform Li stripping/plating and reduced anode‐electrolyte side reactions. In this regard, Sun and co‐workers employed a solution‐based method to fabricate an air‐stable Li_x_SiS_y_ layer on the Li metal surface.^[^ ^63^ ^]^ Thermogravimetric analysis showed that the mass of the Li metal anode with a Li_x_SiS_y_ layer remained constant while the mass of fresh Li metal anode rapidly increased after air‐exposure, demonstrating the air stability of the modified Li metal anode (Figure 2d). The electrochemical results in Figure 2e show that the Li/Li_3_PS_4_/ LiCoO_2_ (LCO) stably operated for only 50 h while the Li‐Li_x_SiS_y_/Li_3_PS_4_/LCO stably cycled for longer. This suggested that the protective layer formed during cycling not only enhanced the air stability of the Li metal anode but also stabilized the Li_3_PS_4_/Li interface (Figure 2e,f).

Ceramic and perovskite materials have also been utilized to stabilize Li metal anodes.^[^ ^64^, ^65^ ^]^ These inorganic layers show a high modulus, as well as excellent moisture, thermal, and chemical stability, enabling them to suppress Li dendrite growth and protect the underlying Li metal from air corrosion. For instance, Yi and co‐workers used spin coating to introduce a fluoride perovskite onto the surface of a Li metal anode, as illustrated in Figure 2g.^[^ ^66^ ^]^ In situ optical images showed that the surface of the Li metal anode remained smooth with a protective layer, providing convincing evidence that the protective layer suppressed Li dendrite growth. The cycling performance of the full cell based on a Li‐KNiF3 anode showed an excellent capacity retention ratio (Figure 2h). Overall, inorganic materials offer several advantages such as a high modulus and excellent chemical stability, but their high mass densities may be a limiting factor. Among candidate inorganic materials, lightweight modified carbon materials with a low electrical conductivity show promise as coating materials for fabricating air‐stable Li metal anodes.

#### 3.1.2 Organic Artificial Layers

Due to the hostless nature of Li metal, Li metal anodes undergo dynamic surface changes during the charge‐discharge process.^[^ ^67^ ^]^ Inorganic layers are mostly used as protective coatings, but they may be damaged over time due to their poor ability to withstand deformation, leading to a loss in their protective function.^[^ ^68^ ^]^ Therefore, protective layers that exhibit excellent self‐adapting capability and air‐stability are required.^[^ ^69^, ^70^ ^]^ Compared with inorganic layers, organic coatings provide many more merits including great diversity, low density, strong adhesion, and good flexibility.^[^ ^71^, ^72^, ^73^ ^]^ Recently, various organic layers have been designed to protect Li metal anodes from air corrosion and stabilize the Li plating‐stripping process.^[^ ^74^, ^75^ ^]^ Based on their molecular size, the used organic molecules can be categorized into either smallmolecules or macromolecules.

**Figure 3:**
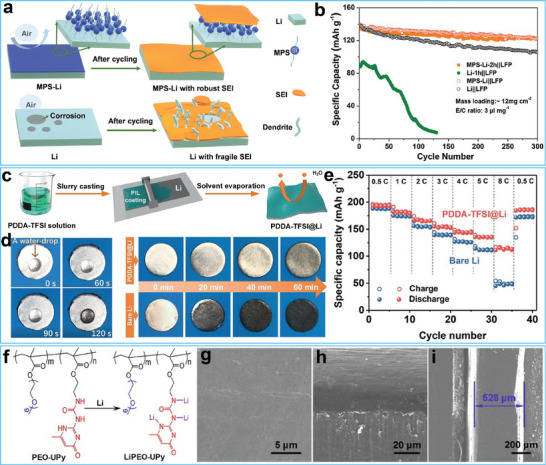
a) Schematic illustrating of the electrochemical plating/stripping processes of 3‐methacryloxypropyltrimethoxysilane (MPS) modified Li metal anode and bare Li metal anode. b) Cycling performance of the batteries based on air‐exposed MPS‐Li metal anode, air‐exposed Li metal anode, MPS‐Li metal anode, and bare Li metal anode. Reproduced with permission.^[^ ^79^ ^]^ Copyright 2021, Wiley‐VCH. c) Preparation process of the artificial SEI layer based on poly (diallyl dimethyl ammonium) (PDDA)‐bis(trifluoromethanesulfonyl)imide (TFSI). d) Water‐stability and air‐stability of the PDDA‐TFSI modified Li metal anode and bare Li metal anode. e) Rate performance of full cell batteries based on PDDA‐TFSI modified Li metal anode and bare Li metal anode. Reproduced with permission.^[^ ^84^ ^]^ Copyright 2021, Wiley‐VCH. f) Molecular structure of poly (ethylene oxide) (PEO)‐ureido‐pyrimidinone (UPy) copolymer and its lithiation reaction process. g–i) SEM images of the LiPEO–UPy modified Li metal anode after 200 cycles. Reproduced with permission.^[^ ^91^ ^]^ Copyright 2020, Wiley‐VCH.

Silane coupling agents are the most commonly used small‐molecule surface modifiers.^[^ ^76^, ^77^, ^78^ ^]^ The inorganic reactive groups of silane coupling agents can react with LiOH to form Li‐O‐Si bonds, while the low‐surface‐energy organic groups provide air tolerance. For example, Guo and co‐workers utilized 3‐methacryloxypropyltrimethoxysilane (MPS) to decorate the Li metal anode to construct a natural solid‐electrolyte interphase (SEI) layer, as illustrated in **Figure** **3**a.^[^ ^79^ ^]^ The low surface energy of silane endowed the modified Li metal anode with excellent air stability. Furthermore, the chemical bond between MPS and Li metal guaranteed strong adhesion between the SEI layer and Li substrate, promoting dense Li deposition. Thus, the fullcell based on an air‐exposed MPS‐Li metal anode exhibited outstanding cycling stability (Figure 3b). Analogues of silane coupling agents have also been employed to functionalize Li metal anodes. In this context, Chen and co‐workers used octadecylphosphonic acid to modify composite microspheres of Li metal and carbon nanotubes.^[^ ^80^ ^]^ The modified composite Li metal anode showed excellent stability in both dry and humid air. Wax is another common material with a low surface free energy, that has been considered a viable option for enhancing the air stability of Li metal anodes.^[^ ^81^ ^]^

Macromolecules are also frequently employed as surface modifiers to functionalize Li metal surfaces. Compared with small organic molecules, macromolecules possess multiple functional groups and exhibit high mechanical strength, allowing them to regulate Li^+^ flux and suppress Li dendrite growth.^[^ ^82^, ^83^ ^]^ In one study, Huang and colleagues employed a polycationic and hydrophobic polymer to modify the Li metal surface using a simple tape‐casting method, as shown in Figure 3c.^[^ ^84^ ^]^ The polymeric cations in the polymer optimized the Li^+^ flux by providing electrostatic shielding, while the anions endowed it with excellent hydrophobicity. Optical images in Figure 3d indicate that the modified Li metal anode could withstand corrosion by humid air and water. The cycling performance of the full‐cell utilizing the modified Li metal anode exhibited excellent rate performance (Figure 3e). Other macromolecules, such as polydimethylsiloxane, polymers of intrinsic microporosity, polyvinyl alcohol, poly(vinylidene‐co‐hexafluoropropylene), ethylene‐vinyl acetate copolymer, poly (arylene ether sulfone)‐g‐poly(ethylene glycol) copolymer, and polyurethane elastomers, have also been used to functionalize the Li metal surface, resulting in improved air stability.^[^ ^85^, ^86^, ^87^, ^88^, ^89^, ^90^ ^]^ In addition to air stability, Li ion conduction is another crucial characteristic of organic protective layers. Hence, Xiong and co‐workers synthesized a copolymer that incorporated poly(ethylene oxide) (PEO) segments and ureido‐pyrimidinone (UPy) moieties (Figure 3f).^[^ ^91^ ^]^ The PEO‐UPy copolymer was further adopted as a protective layer to decorate the Li metal anode. The PEO‐UPy coating layer exhibited excellent air stability, and the introduction of the PEO segment gave the layer a high Li ion conductivity (2.37×10^–5^ S cm^–5^). As a result, the modified Li metal anode exhibited a dense structure and smooth surface (Figure 3g–i).

#### 3.1.3 Organic‐Inorganic Hybrid Layers

Inorganic and organic layers each have their own advantages and advantages.^[^ ^92^ ^]^ In terms of modulus, inorganic layers have an advantage over organic layers, but they lack sufficient toughness, while organic layers have good toughness but lower modulus.^[^ ^93^, ^94^ ^]^ Therefore, constructing hybrid layers that combine both inorganic and organic materials is a logical choice for Li metal anodes.^[^ ^95^, ^96^, ^97^ ^]^ The high modulus of the inorganic component can suppress Li dendrite growth, while the excellent flexibility of the organic component helps maintain the structural integrity of composite coatings. Additionally, the surface of inorganic nanoparticles can be modified with low‐surface‐energy materials to change their surface from hydrophilic to hydrophobic. This hydrophobicity of the nanoparticles enhances the overall hydrophobicity of the hybrid layers, thus improving the air stability of composite Li metal anodes. Furthermore, some inorganic nanoparticles, such as ceramic electrolytes, exhibit excellent Li^+^ conductivity, and introducing nanoparticles into the organic moiety can enhance the Li^+^ conductivity of the hybrid layers.

In this regard, Lu and co‐workers utilized a simple vapor deposition technique to construct organic–inorganic coatings on a Li metal surface.^[^ ^98^ ^]^ Using a mixed vapor of 3‐mercaptopropyl trimethoxysilane (MPS) and tetraethoxysilane (TEOS). The incorporation of the organic moiety MPS rendered the composite coating highly flexible and tough. Additionally, the hydrolysis and condensation reactions resulted in the generation of Li_x_SiO_y_, thus improving the mechanical properties of the hybrid coating. Due to the exceptional anti‐corrosion ability of phosphates, Yan and co‐workers developed an organic–inorganic interface on the surface of Li metal by introducing polyphosphoric ester as a modifier.^[^ ^99^ ^]^ A dual‐layer interface was created using a surface chemistry approach, with Li_3_PO_4_ as the bottom layer and ‐COPO_3_‐, ‐(CO)_2_PO_2_‐, and‐(CO)_3_PO‐ as the upper layer. Xie and co‐workers used an in situ chemical reaction to fabricate an organic‐inorganic polymer‐alloy hybrid layer thatimparted excellent air stability and long‐term cycling to the Li metal anode.

**Figure 4:**
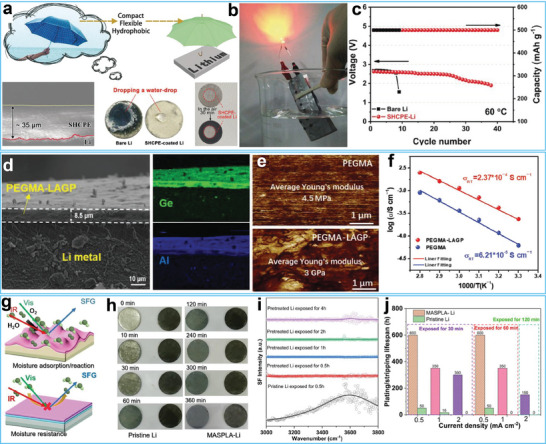
a) Schematic illustration of the protective effect of the hydrophobic layer on Li metal anode, the thickness of the layer, the water resistance and air stability of the as‐formed layer. b) the underwater test of the Li‐air battery based on the hydrophobic composite polymer electrolyte. c) Cycling performance of the Li‐air battery with and without hydrophobic composite polymer electrolyte at 60 °C. Reproduced with permission.^[^ ^102^ ^]^ Copyright 2019, Wiley‐VCH. d) SEM image of the Li metal anode with PEGMA‐LAGP layer. e) Atomic force microscope (AFM) test of the pristine PEGMA layer and PEGMA‐LAGP layer. f) Li ion conductivity of the pristine PEGMA layer and PEGMA‐LAGP layer. Reproduced with permission.^[^ ^103^ ^]^ Copyright 2022, Wiley‐VCH. g) Schematic illustration of the moisture‐stability of the pristine Li metal anode and modified Li metal anode. h) Air‐exposing test of the pristine Li metal anode and modified Li metal anode with various exposing time. i) Interface‐sensitive SFG spectroscopy result of the pristine Li metal anode and modified Li metal anode with various exposing time. j) Cycling life of the cells based on pristine Li metal anode and modified Li metal anode with various exposing time. Reproduced with permission.^[^ ^104^ ^]^ Copyright 2022, Elsevier.

In addition to air stability, the hybrid layer must have a high Li‐ion conductivity to assemble high‐rate batteries or all‐solid‐state batteries.^[^ ^100^, ^101^ ^]^ Recently, Zhang and co‐workers developed a hydrophobic composite polymer electrolyte by incorporating hydrophobic SiO_2_ nanoparticles into a high ionic‐conductive thermoplastic polyurethane.^[^ ^102^ ^]^ The as‐prepared hydrophobic composite polymer electrolyte protected the Li metal anode from corrosion by water and air, as illustrated in **Figure** **4**a. Due to the excellent hydrophobicity of the hybrid polymer electrolyte, the assembled Li‐air battery lit up a red LED, even after immersion in water (Figure 4b). Additionally, the hybrid polymer electrolyte also showed excellent heat resistance, giving the Li‐air battery a longer cycle life (Figure 4c). Wu and co‐workers reported the synthesis of a flexible organic–inorganic hybrid electrolyte with a thickness of 8.5 µm using a thermal‐initiated free‐radical reaction (Figure 4d).^[^ ^103^ ^]^ The inclusion of nanoparticles significantly enhanced the mechanical properties of poly(ethylene glycol methacrylate) and increased its Young's modulus from 4.5 MPa to 3 GPa (Figure 4e). Moreover, the Li_1.5_Al_0.5_Ge_1.5_(PO_4_)_3_ nanoparticles were rapid ion conductors, giving the hybrid material a high ionic conductivity (2.37 × 10^−4^ S cm^−1^; Figure 4f). This finding highlights the potential of hybrid electrolytes for all‐solid‐state lithium batteries. Li and co‐workers developed a spin‐coating method to create an artificial SEI layer on Li metal surfaces.^[^ ^104^ ^]^ This SEI layer was composed of a mixture of tin difluoride (SnF_2_), poly(vinylidenecfluoride‐*co*‐hexafluoropropene) (PVDF‐HFP), and dimethyl sulfoxide (DMSO). The resulting SEI layer consisted of a LiF/Sn/Li_x_Sn alloy layer that increased the mechanical properties and ion conductivity of the SEI layer. The PVDF‐HFP layer ensured the flexibility and transport of ions within the SEI layer. The low surface energy of the PVDF‐HFP also contributed to the excellent moisture resistance of the SEI layer, as presented in Figure 4g,h. The moisture resistance was further supported by interface‐sensitive and interface‐selective sum frequency generation (SFG) spectroscopy, as shown in Figure 4i. Figure 4j illustrates the electrochemical properties, demonstrating that when equipped with a well‐designed SEI layer, the Li metal anode maintained its long‐term cycling even when exposed to humid air. Overall, organic‐inorganic hybrid layers exhibit a favorable combination of strength and toughness, making them the most promising choice for Li metal anode coatings.

#### 3.1.4 Composite Li Metal Anodes

Different from the intercalation mechanism of graphite anodes, Li metal anodes undergo a continuous plating/stripping process during cycling.^[^ ^105^ ^]^ The plating/stripping process, along with the hostless nature, results in infinite relative volume changes during cycling.^[^ ^106^, ^107^ ^]^ To address this issue, carbon materials, metals, and other conductive materials have been developed as hosts.^[^ ^108^, ^109^, ^110^ ^]^ Thus, it is reasonable to design composite Li metal anodes for practical applications to also reduce the exposure of Li metal to air and therefore improve its air stability.^[^ ^111^, ^112^, ^113^ ^]^

**Figure 5:**
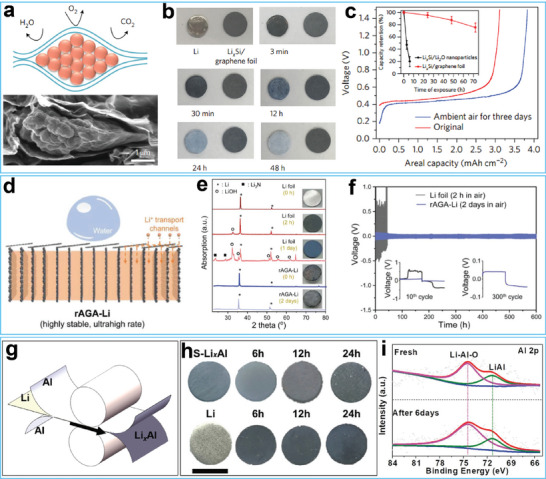
a) Schematic illustration of Li_x_M/graphene foil. b) Photographs of pristine Li foil and Li_x_Si/graphene foil after air‐exposing test. c) Areal capacity retention of the LixSi/graphene foil before and after air‐exposing. Reproduced with permission.^[^ ^114^ ^]^ Copyright 2017, Springer Nature. d) Schematic illustration of water‐stable composite Li metal anode composing reduced by accordion‐like graphene oxide array (rAGA) and Li foil. e) XRD spectra of pristine Li metal anode and rAGA‐Li anode after air‐exposing with various time. f) Cycling test of symmetrical cells assembling by two air‐treated samples. Reproduced with permission.^[^ ^115^ ^]^ Copyright 2020, Wiley‐VCH. g) Schematic of preparation process of Li‐Al alloy foil by roll‐to‐roll technology. h) Photographs of pristine Li foil and Li‐Al alloy foil after various air‐exposing times. i) XPS spectra of Li‐Al alloy foil before and after 6 days air‐exposing. Reproduced with permission.^[^ ^116^ ^]^ Copyright 2021, Wiley‐VCH.

To enhance the air stability and alleviate volume changes of Li metal anodes, Cui and co‐workers developed a composite Li metal anode by encapsulating Li‐containing nanoparticles into graphene sheets, as displayed in **Figure** **5**a.^[^ ^114^ ^]^ Due to the protection of graphene sheets, the composite Li metal anode exhibited excellent air stability, as shown by the photographs during air exposure experiments (Figure 5b). Electrochemical tests showed that the areal capacity of the graphene‐wrapped anode was only slightly deteriorated after exposure to the ambient air, in contrast to the sharp capacity decline in the unprotected anode after air corrosion (Figure 5c). Liu and co‐workers designed a dual‐layer host composed of aligned graphene sheets at the base and sloping‐aligned graphene sheets on top (Figure 5d).^[^ ^115^ ^]^ The bottom layer of the aligned graphene sheets served as a reservoir for Li ions, while the surface layer of graphene sheets protected metallic Li from air corrosion. In the optical photograph in Figure 5e, the color of the graphene‐based composite Li metal anode remained relatively constant after two days of exposure, whereas the pristine Li foil quickly turned black from its original silver‐like color. Electrochemical tests of symmetrical cells demonstrated that the composite Li metal anode exhibited stable long‐term cycling and a low overpotential of ≈50 mV, even after being exposed to the air for two days. In contrast, the pristine Li foil hardly cycled, even after only two hours of exposure to the air (Figure 5f). Li and co‐workers adopted an air‐stable Al foil to protect the Li metal anode.^[^ ^116^ ^]^ By employing a roll‐to‐roll mechanical prelithiation method, they fabricated a Li_x_Al alloy anode (Figure 5g). The color changes in Figure 5h demonstrated the air stability of the as‐prepared Li_x_Al alloy foil. X‐ray photoelectron spectroscopy (XPS) test revealed the protective layer of the Li_x_Al alloy (Figure 5i), revealing the presence of Li‐Al‐O compounds on the surface of the Li_x_Al in both the freshly‐prepared sample and the sample exposed to humid air for six days. These compounds functioned as a passivation layer that protected the Li_x_Al alloy from corrosion by humid air.

Numerous attempts have been proposed to develop air‐stable Li metal anodes, including organic layers, inorganic layers, organic‐inorganic hybrid layers, and composite designs. The main strategy for protecting Li metal anodes from air‐corrosion involves decorating the surface of Li metal anode with hydrophobic materials or chemically‐inert materials. Increasing the roughness of the Li metal surface can enhance its hydrophobicity, but this has received little attention because it requires complex pretreatment techniques. Using lithium powder to construct air‐stable Li metal anodes may be a practical option. The presence of voids in powder particles allows for the convenient construction of rough surfaces. Thus, a reasonable approach for preparing air‐stable Li metal anodes may involve functionalizing Li powder with suitable surface modifiers and then coating it onto a conductive substrate.

### 3.2 Solid‐State Electrolytes

The liquid electrolytes commonly used in Li metal batteries have shown advantages in terms of rapid Li^+^ transportation and excellent interfacial contact with anode/cathode, but their flammability, propensity for explosion, and toxicity have limited their further applications.^[^ ^117^, ^118^, ^119^ ^]^ SSEs have emerged as a viable strategy because their non‐flammability ensures the safe operation of fully assembled batteries.^[^ ^120^, ^121^, ^122^ ^]^ Additionally, the higher modulus of SSEs enables them to inhibit the growth of Li dendrites, which prolongs the lifespan of Li metal batteries. Previously, many SSEs have been reported, including polymer‐based SSEs, oxide‐based SSEs, sulfide‐based SSEs, and halide‐based SSEs.^[^ ^123^, ^124^ ^]^ Among them, polymer‐based SSEs always exhibit superior air stability. Due to their high surface chemical reactivity, the other three types of SSEs exhibit air instability, posing a challenge to their application.^[^ ^125^, ^126^, ^127^, ^128^ ^]^

#### 3.2.1 Oxide‐Based Solid‐State Electrolytes

Certain oxide‐based SSEs show excellent stability in ambient air, but other oxide‐based SSEs, such as cubic garnet‐based Li_7_La_3_Zr_2_O_12_ (LLZO) and its variants, are susceptible to chemical reactions with moisture in the air.^[^ ^129^ ^]^ These reactions form by‐products that cover the surface.^[^ ^130^, ^131^ ^]^ This uncontrollable formation of lithiophobic species such as Li_2_CO_3_ and LiOH hinders the wetting of LLZO by metallic Li, leading to an uneven Li^+^ flux distribution during Li plating/stripping, which in turn contributes to the nucleation and growth of dendrites throughout the bulk electrolyte. These dendrites may ultimately cause internal short circuits in batteries.^[^ ^132^ ^]^ Some studies have reported that elemental doping can enhance the air stability of LLZO to some degree, but the air instability of LLZO and its derivatives persists.^[^ ^133^, ^134^ ^]^

**Figure 6:**
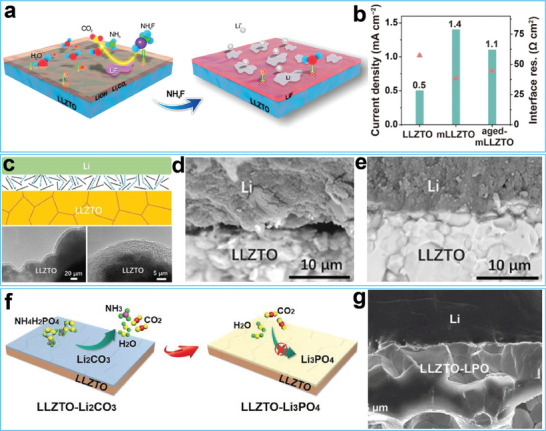
a) Schematic illustration of the surface treatment process by adopting ammonium fluoride (NH_4_F). b) Critical current density and interfacial resistance of the Li/Li symmetric cells based on the Li_6.4_La_3_Zr_1.4_Ta_0.6_O_12_, modified Li_6.4_La_3_Zr_1.4_Ta_0.6_O_12_ before and after aging. Reproduced with permission.^[^ ^138^ ^]^ Copyright 2020, Wiley‐VCH. c) Diagram of the LGC lithiation process and TEM images of LGC coated Li_6.75_La_3_Zr_1.75_Ta_0.25_O_12_. d, e) SEM images of the Li/Li_6.75_La_3_Zr_1.75_Ta_0.25_O_12_ interface without and with LGC layer. Reproduced with permission.^[^ ^139^ ^]^ Copyright 2020, Wiley‐VCH. f) Schematic illustration of the surface treatment process by adopting NH_4_H_2_PO_4_ salt. g) SEM images of the Li/ Li_6.4_La_3_Zr_1.4_Ta_0.6_O_12_ interface with Li_3_PO_4_ layer after melting. Reproduced with permission.^[^ ^140^ ^]^ Copyright 2022, Wiley‐VCH.

Surface decoration can also be used to protect garnet‐based SSEs.^[^ ^135^, ^136^ ^]^ Because the surface of SSEs is already contaminated with lithiophobic species, these contaminants should be removed before surface decoration. In this study, Arava and co‐workers first purified the surface of Li_6.5_La_3_Zr_1.5_Ta_0.5_O_12_ by annealing it at high temperature under an ultrahigh vacuum conditions and then coated a nanometer‐thick h‐BN layer onto the Li_6.5_La_3_Zr_1.5_Ta_0.5_O_12_ surface using atomic layer deposition.^[^ ^137^ ^]^ In another study, Guo and co‐workers developed a moderate‐temperature conversion chemistry method for treating the surface of Li_6.4_La_3_Zr_1.4_Ta_0.6_O_12_ with ammonium fluoride (NH_4_F), as illustrated in **Figure** **6**a.^[^ ^138^ ^]^ At a moderate temperature, the surface contaminants on Li_6.4_La_3_Zr_1.4_Ta_0.6_O_12_ reacted with NH_4_F and formed a fluorinated nano‐coating. The resultant low surface energy of the fluoride coating rendered the modified garnet surface hydrophobic, thus preventing air corrosion upon re‐exposure to the air. Additional experimental results demonstrated that the modified Li_6.4_La_3_Zr_1.4_Ta_0.6_O_12_ showed reduced interfacial resistance and increased critical current density before and after aging compared with the unmodified Li_6.4_La_3_Zr_1.4_Ta_0.6_O_12_ (Figure 6b). Xia and co‐workers reported the concurrent removal of surface lithiophobic contaminants and the protection of Li_6.75_La_3_Zr_1.75_Ta_0.25_O_12_ against air and moisture.^[^ ^139^ ^]^ This was achieved by depositing a low‐graphitized carbon (LGC) layer on its surface using a thermal‐decomposition vapor deposition method, as shown in Figure 6c. The introduction of the LGC layer enhanced the air‐stability of the modified Li_6.75_La_3_Zr_1.75_Ta_0.25_O_12_ and also improved its Li wettability after lithiation, as demonstrated by the SEM images in Figure 6d,e. Guo and co‐workers purified the surface of Li_6.4_La_3_Zr_1.4_Ta_0.6_O_12_ and simultaneously maintained its air stability by utilizing the conversion reaction between molten NH_4_H_2_PO_4_ salt and Li_2_CO_3_ (Figure 6f).^[^ ^140^ ^]^ The resulting Li_3_PO_4_ layer acted as a barrier against moisture attack. The SEM images in Figure 6g also show that during the melt infiltration process, the Li_3_PO_4_ layer transformed into a lithiophilic Li_3_P/Li_2_O interlayer, creating smooth and seamless contact between Li and Li_6.4_La_3_Zr_1.4_Ta_0.6_O_12_.

#### 3.2.2 Sulfide‐Based Solid‐State Electrolytes

Compared with oxide‐based SSEs, sulfide‐based SSEs have higher Li^+^ conductivities (from 10^−4^ to 10^−2^ S cm^−1^) at room temperature due to the larger ionic radii and weaker electronegativity of sulfide ions.^[^ ^141^, ^142^ ^]^ In addition, sulfide‐based SSEs possess wide electrochemical windows and negligible electronic conductivities. Notably, the grain boundary resistance of sulfide‐based SSEs can be significantly reduced by cold‐pressing at room temperature, which eliminates the need for high‐temperature sintering. However, the large‐scale application of sulfide‐based SSEs is limited due to their sensitivity to humid air.^[^ ^143^ ^]^ The air‐instability of sulfide‐based SSEs is attributed to the high chemical activity of P S contaminants. According to the hard and soft acids and bases theory,^[^ ^144^ ^]^ the hard acid P in Li_3_PS_4_ tends to react with oxygen in air/moisture, forming P‐O bonds and disrupting the original P‐S bonded structure. The soft base property of sulfur atoms causes them to react with hydrogen atoms of water molecules in the air, resulting in the release of H_2_S gas. Soft acids and soft bases tend to form stable complexes. Therefore, many soft acids, such as Ge, Sb, Zn, Cu, and Sn, and others, have been selected as co‐dopants with P.^[^ ^145^, ^146^, ^147^, ^148^, ^149^ ^]^ The introduced dopants can bond with S, thus preventing the formation of H_2_S when exposed to the air. Co‐doping Li_3_PS_4_ with a metallic element and oxygen is also feasible, as oxide‐based SSEs are more air‐stable than sulfide‐based SSEs.^[^ ^150^, ^151^ ^]^

**Figure 7:**
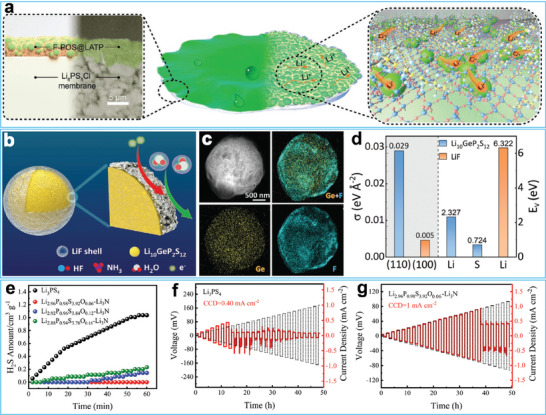
a) Hydrophobic surface fabricated by fluorinated polysiloxane coated Li_1.4_Al_0.4_Ti_1.6_(PO_4_)_3_ (LATP) nanoparticles. Reproduced with permission.^[^ ^153^ ^]^ Copyright 2022, Wiley‐VCH. b) Schematic illustration of the core‐shell SSE. c) Elemental mapping images of the as‐prepared core‐shell SSE. d) Surface energies (*σ*) and vacancy formation energies (*E_v_*) for Li_10_GeP_2_S_12_ and LiF. Reproduced with permission.^[^ ^154^ ^]^ Copyright 2023, Wiley‐VCH. e) H_2_S gas emission curves of Li_3_PS_4_ before and after element doping. f,g) Critical current density (CCD) of the Li–Li cells assembled with Li_3_PS_4_ and element doped Li_3_PS_4_. Reproduced with permission.^[^ ^155^ ^]^ Copyright 2022, Wiley‐VCH.

Another feasible strategy for enhancing the air stability of sulfide‐based SSEs is to decorate their surface with a protective layer.^[^ ^152^ ^]^ In this regard, Wu and co‐workers introduced a superhydrophobic Li^+^‐conducting coating layer on the surface of a sulfide‐based SSE.^[^ ^153^ ^]^ The coating layer was prepared by using Li_1.4_Al_0.4_Ti_1.6_(PO_4_)_3_ (LATP), tetraethyl orthosilicate, and 1*H*,1*H*,2*H*,2*H*‐perfluorodecyltriethoxysilane (PFDTES) as the raw materials via hydrolysis and condensation. The prepared fluorinated polysiloxane‐coated LATP nanoparticles showed excellent water resistance (**Figure** **7**a). Yao and co‐workers utilized a gas‐phase treatment method to decoratethe surface of Li_10_GeP_2_S_12_ with a LiF layer (Figure 7b).^[^ ^154^ ^]^ The uniformity of the coating on the Li_10_GeP_2_S_12_ surface was confirmed through element maps (Figure 7c). The LiF‐coated SSE demonstrated superior air stability due to the ultra‐low surface energy (0.005 eV) of LiF (Figure 7d). Yang and co‐workers in situ constructed a Li_3_N layer on the surface of Li_3_PS_4_ by using O and N as co‐dopants.^[^ ^155^ ^]^ Due to the synergistic effect between the Li_3_N layer and POS_3_^3−^, the modified SSE suppressed the generation of H_2_S gas (Figure 7e). The Li_3_N by‐product passivated the interface between Li and the solid electrolyte, thus enhancing the critical current density from 0.40 to 1.00 mA cm^−2^ (Figure 7f,g).

#### 3.2.3 Halide‐Based Solid‐State Electrolytes

Halide‐based SSEs have simple synthesis, high Li^+^ conductivity, and mechanical softness.^[^ ^156^, ^157^ ^]^ Their excellent Li^+^ conductivity is due to the larger ionic radius of univalent halogen ions and weaker interatomic forces between univalent halogen ions and lithium ions. Their mechanical softness endows them with excellent interfacial compatibility with high‐voltage cathode materials. Many well‐designed halide‐based SSEs are used to construct high‐performance SSLMBs, but the poor air stability of halide‐based SSEs arising from the moisture hypersensitivity of halide salts hampers their further applications.^[^ ^158^, ^159^ ^]^

**Figure 8:**
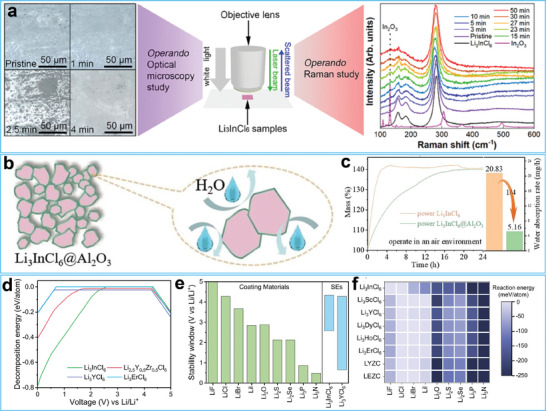
a) Schematic illustration of the *operando* characterization method and Raman spectroscopy setup adopted in investigating Li_3_InCl_6_ samples (middle), the *operando* optical images of Li_3_InCl_6_ samples with different exposing time in air with 30% humidity (left), and the Raman spectra evolution of Li_3_InCl_6_ samples (right). Reproduced with permission.^[^ ^160^ ^]^ Copyright 2020, American Chemical Society. b) Schematic illustration of the Al_2_O_3_ coated Li_3_InCl_6_ samples. c) Mass‐time curves and water absorption rate of Li_3_InCl_6_ and modified Li_3_InCl_6_ samples. Reproduced with permission.^[^ ^161^ ^]^ Copyright 2022, Wiley‐VCH. d) The decomposition energy values of certain halide‐based SSEs, e) the electrochemical stability window of coating materials for the anode‐electrolyte interface, and f) the reaction energy between coating materials and chloride‐based SSEs based on first‐principles calculations. Reproduced with permission.^[^ ^162^ ^]^ Copyright 2022, American Chemical Society.

To solve this issue, researchers have explored the underlying mechanism responsible for the air‐instability of halide‐based SSEs to propose effective solutions. For example, Sun and co‐workers studied the degradation of Li_3_InCl_6_ at different humidity levels by adopting ex situ, in situ, and operando characterization methods.^[^ ^160^ ^]^ Their results confirmed that Li_3_InCl_6_ showed excellent air stability in a low‐humidity environment (3%). Once exposed to a high humidity, Li_3_InCl_6_ first transformed into crystalline hydrates and then degraded into In_2_O_3_, LiCl, and HCl (**Figure** **8**a), leading to a performance degradation. To improve the air stability, Li and co‐workers decorated the surface of Li_3_InCl_6_ with Al_2_O_3_ by powder atomic layer deposition (Figure 8b).^[^ ^161^ ^]^ The mass‐time curves in Figure 8c show that the Al_2_O_3_ layer inhibited the water adsorption of modified Li_3_InCl_6_. Using first‐principles calculations, Yu and co‐workers calculated the decomposition energy of some halide‐based SSEs (Figure 8d), the electrochemical stability window of coatings for the anode‐electrolyte interface (Figure 8e), and the reaction energy between coating materials and chloride‐based SSEs (Figure 8f).^[^ ^162^ ^]^ The results demonstrated that coatings containing binary halides stabilized the interface between Li metal anode and halide‐based SSEs. These results will also help guide the design of air‐stable coating layers for halide‐based SSEs.

### 3.3 High‐Energy Cathodes

As a crucial component of solid‐state lithium metal batteries, cathodes make a critical contribution to the high energy output of fully‐assembled batteries.^[^ ^163^ ^]^ Nickel‐rich layered oxides (LiNi_x_Co_y_Mn_1‐x‐y_O_2_, x ≥ 0.6) and lithium sulfide (Li_2_S) cathodes have gained significant attention and are widely used in this regard.^[^ ^164^, ^165^ ^]^ Increasing the Ni content in the nickel‐rich layered oxides can improvethe specific capacity, but when the nickel content exceeds 70%, significant side reactions occur between the cathodes and surrounding air.^[^ ^166^, ^167^ ^]^ These side reactions form by‐products, such as LiOH, Li_2_CO_3_, and LiHCO_3_, commonly referred to as “residual lithium”. For Li_2_S cathodes, the intrinsic reaction between Li_2_S and H_2_O deteriorates the Li_2_S cathode and also releases toxic H_2_S gas.^[^ ^168^ ^]^ Therefore, there is a need to increase the air stability of toxic high‐energy cathodes.

#### 3.3.1 Nickel‐Rich Layered Oxides

The air‐instability of nickel‐rich layered oxides is primarily attributed to two factors. The first is the redox reactions of nickel‐rich layered oxides. According to the crystal field theory, the presence of Ni^3+^ always induces Jahn‐Teller distortion in nickel‐rich layered oxides, and Ni^3+^ tends to be reduced to Ni^2+^ to prevent this structural evolution. This reduction process generates active oxygen species that further react with CO_2_ and H_2_O and forma residual lithium layer on the cathode surface. The second reason is that H^+^/Li^+^ exchange always occurs. When a nickel‐rich layered oxide contacts humid air, Li^+^ ions tend to migrate outward, while H^+^ ions tend to diffuse into the bulk phase, leading to the generation of harmful residual lithium that can negatively impact the electrode fabrication process and cycling stability. Elemental doping is adopted to improve the air stability of nickel‐rich layered cathodes.^[^ ^169^ ^]^ Cationic dopants can occupy the transition metal sites and form stronger metal–oxygen bonds, thus enhancing the structural stability of the modified cathodes.^[^ ^170^ ^]^

**Figure 9:**
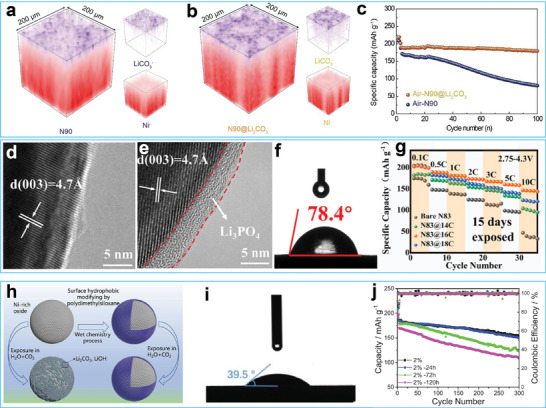
a,b) 3D ToF‐SIMS images of the high‐Ni cathode before and after CO_2_ treatment. c) Cycling performance of the fresh high‐Ni cathode and high‐Ni oxide cathode with dense Li_2_CO_3_ layer after air‐exposing. Reproduced with permission.^[^ ^172^ ^]^ Copyright 2022, Wiley‐VCH. d,e) TEM images of high‐Ni cathode before and after surface treatment with alkyl phosphoric. f) Water contact angle of the modified sample. g) Rate performance of the modified samples. Reproduced with permission.^[^ ^173^ ^]^ Copyright 2023, Elsevier. h) Schematic illustration of the coating process with polydimethylsiloxane. i) Water contact angle of polydimethylsiloxane modified sample. j) Cycle performance of polydimethylsiloxane modified sample with various exposing time in a damp chamber at 50% relative humidity and 25°C. Reproduced with permission.^[^ ^174^ ^]^ Copyright 2022, Wiley‐VCH.

Surface coatings have also been utilized to enhance the air stability of high‐nickel cathodes.^[^ ^171^ ^]^ In this regard, Guo and co‐workers improved the cathode surface by creating a dense layer of Li_2_CO_3_ using a CO_2_ treatment process.^[^ ^172^ ^]^ The 3D ToF‐SIMS images in **Figure** **9**a,b show that the Li_2_CO_3_ layer was evenly dispersed on the cathode surface. The experimental results confirmed that the fabricated Li_2_CO_3_ layer enhanced the air stability of the high‐nickel cathode due to its thermodynamic stability. Additionally, the Li_2_CO_3_ layer consumed the generated HF during cycling and transformed into a robust fluorine‐containing cathode electrolyte interphase (CEI). Consequently, the modified cathode with a dense Li_2_CO_3_ layer exhibited more stable cycling performance than the fresh cathode, even after exposure to air (Figure 9c). Zheng and co‐workers adopted alkyl phosphoric to decorate the surface of LiNi_0.83_Mn_0.11_Co_0.06_O_2_ to fabricate a hydrophobic coating containing inorganic and organic components (Figure 9d–f).^[^ ^173^ ^]^ The inorganic lithium phosphate prevented electrolyte corrosion and accelerated lithium‐ion transport, while the low surface energy organic component endowed it with hydrophobicity, thus alleviating Li^+^/H^+^ ion exchange once exposing to the air. Due to the synergistic protective effect of the lithium phosphate and hydrophobic coating, the modified LiNi_0.83_Mn_0.11_Co_0.06_O_2_ demonstrated superior rate capability to the fresh samples after 15 days of exposure to the air (Figure 9g). Qiu and co‐workers coated polydimethylsiloxane on the surface of LiNi_0.8_Co_0.1_Mn_0.1_O_2_ particles to enhance their hydrophobicity (Figure 9h,i).^[^ ^174^ ^]^ The enhanced hydrophobicity improved their air corrosion resistance, allowing the modified samples to withstand 24 h of exposure in a damp chamber at 50% relative humidity at 25°C (Figure 9j).

#### 3.3.2 Lithium Sulfide

Lithium sulfide (Li_2_S) is a better candidate for next‐generation lithium‐ion batteries due to several advantages.^[^ ^175^, ^176^ ^]^ First, lithium sulfide has an outstanding theoretical capacity (1166 mA h g^−1^), which is significantly higher than that of existing commercial cathode materials. Lithium sulfide also has a low density (1.66 g cm^−3^), which can minimize the volume expansion of the cathode during cycling. More importantly, lithium sulfide can be used to assemble lithium‐free anodes to avoid safety concerns arising from Li metal anodes. Although promising, the applications of lithium sulfide are still immature due to their poor electronic and ionic conductivities, polysulfide shuttling, large activation barrier, and air instability.^[^ ^177^, ^178^ ^]^ In Particular, they suffer from the issue of air instability and can release toxic H_2_S once exposed to an ambient environment.

**Figure 10:**
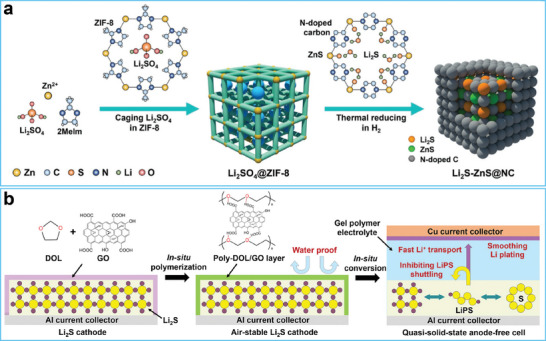
a) Sketch map of the synthetic process of carbon cage encapsulate Li_2_S cathode. Reproduced with permission.^[^ ^181^ ^]^ Copyright 2019, Wiley‐VCH. b) Schematic illustration of the preparation process of the air‐stable composite layer toward Li_2_S cathode. Reproduced with permission.^[^ ^182^ ^]^ Copyright 2023, Wiley‐VCH.

To improve the air stability of lithium sulfide, researchers have tried encapsulated it into a host or wrapped it with a coating.^[^ ^179^, ^180^ ^]^ For example, Qiu and co‐workers designed and prepared a N‐doped carbon cage for lithium sulfide (**Figure** **10**a).^[^ ^181^ ^]^ Due to confinement in the cage, the lithium sulfides showed improved stability, kinetics, and reversibility. Wang and co‐workers utilized a dense layer consisting of poly‐1,3‐dioxolane (DOL) and graphene oxide (GO) to protect the Li_2_S cathode from air corrosion, which improved the air stability of the polymer‐wrapped lithium sulfide. (Figure 10b).^[^ ^182^ ^]^ Furthermore, this protective layer transformed into a gel electrolyte when liquid electrolyte was incorporated, which further inhibited the shutting of lithium polysulfides and smoothed the Li metal anode.

## 4 Summary and Outlook

Solid‐state lithium metal batteries have been recognized as promising energy storage devices for the near future, but their key materials, such as Li metal anodes, SSEs, and high‐energy cathodes, exhibit inferior air stability, which leads to a variety of performance issues and even device failure. Enhancing the air stability of the battery materials has several advantages, including reduced battery material storage costs, streamlined manufacturing processes, and reduced safety risks when exposed to the air. Therefore, it is crucial to design air‐stable battery materials, which can be accomplished by constructing well‐designed protective layers. In this manuscript, we provided a comprehensive review of research progress in improving the air stability of battery materials, and the protective mechanisms involved by focusing on the Li metal anodes, SSEs, and high‐energy cathodes.

The development of air‐stable battery materials has been inspired bylotus leaves. To create hydrophobic surfaces, coating materials with a low surface energy are needed. Many fluorine‐containing materials, including inorganic and organic materials, have been designed, synthesized, and wrapped around battery materials to act as protective layers, thus changing the surface of battery materials from hydrophilic to hydrophobic. The surface hydrophobicity isolates the battery materials from moisture, thus avoiding of water corrosion. Additionally, other air‐stable materials, such as carbon materials, alloys, and ceramics, have also been adopted to protect the battery materials by reducing the contact area with humid air. These strategies have improved the air stability of battery materials to some extent, but novel protection mechanisms and methods are still needed to directly design and prepare macroscopic air‐stable battery materials.

Novel ideas for constructing hydrophobic surfaces require an understanding of the protective mechanism. Drawing inspiration from the lotus leaf, a low surface energy and high surface roughness are the two key factors in creating superhydrophobic surfaces. Previous studies mainly focused on reducing the surface energy of battery materials by decorating them with low surface energy materials, while surface roughness has received little attention. In future research, attention should also be paid to enhancing the surface roughness of battery materials. The combination of a lower surface energy and greater surface roughness should be considered.

In addition to the lotus leaf, the *Nepenthes* pitcher plant may also provide inspiration for biomimetic surface coatings.^[^ ^183^ ^]^ Different from storing an air layer in the microstructure of plant surfaces to adjust the surface wettability, the *Nepenthes* pitcher plant adjusts its surface wettability by locking an intermediary liquid in the micro‐textures of its pitcher organs. This forms a slippery liquid‐infused porous surface. Following this concept, researchers have attempted to fabricate slippery surfaces by infusing lubricating fluids into a roughened surface.^[^ ^184^ ^]^ This slippery surface repels some liquids that are immiscible with the lubricating fluid, which offers a new pathway for constructing super‐hydrophobic surfaces. Compared with the super‐hydrophobic surface based on the lotus effect, this slippery surface is stable and easy to control. This phenomenon presents a remarkably simple alternative for fabricating hydrophobic surfaces, and may provide valuable insights into the fabrication of air‐stable battery materials.

When designing protective layers, the following key parameters should consider: hydrophobicity, durability, anti‐fatigue performance, ionic transport, and electrochemical stability. Currently, few protective layers meet all these requirements. Future work should extend the functionality of protective layers, and it is also necessary to develop a hydrophobic protective layer with excellent flame‐retardancy to further enhance the safety of batteries. Apart from material selection, the coating technology used to fabricate the hydrophobic layers on battery material surfaces is also crucial to consider. Solvent‐free methods show promise and should be further investigated.

In summary, the air stability of battery materials is a critical issue during the fabrication of solid‐state batteries and can be enhanced using surface coating., and the exploration of novel battery materials with excellent air stability will expedite the commercialization of solid‐state batteries.

## Conflict of Interest

The authors declare no conflict of interest.

## References

[1] J.‐M. Tarascon , M. Armand , Nature 2001, 414, 359. 11713543 10.1038/35104644

[2] S. Xin , Z. Chang , X. Zhang , Y.u‐G. Guo , Nat. Sci. Rev. 2017, 4, 54.

[3] A. Hu , W. Chen , F. Li , M. He , D. Chen , Y. Li , J. Zhu , Y. Yan , J. Long , Y. Hu , T. Lei , B. Li , X. Wang , J. Xiong , Adv. Mater. 2023, 2304762.

[4] C. Zhao , Z. Yan , B. Zhou , Y. Pan , A. Hu , M. He , J. Liu , J. Long , Angew. Chem., Int. Ed. 2023, 62, e202302746. 10.1002/anie.202302746 37300514

[5] S. Xin , Y. You , S. Wang , H.‐C. Gao , Y.a‐X. Yin , Y.u‐G. Guo , ACS Energy Lett. 2017, 2, 1385.

[6] Y. Tang , L. Zhang , J. Chen , H. Sun , T. Yang , Q. Liu , Q. Huang , T. Zhu , J. Huang , Energy Environ. Sci. 2021, 14, 602.

[7] Y.‐G. Lee , S. Fujiki , C. Jung , N. Suzuki , N. Yashiro , R. Omoda , D.‐S. Ko , T. Shiratsuchi , T. Sugimoto , S. Ryu , J. H. Ku , T. Watanabe , Y. Park , Y. Aihara , D. Im , I. T. Han , Nat. Energy 2020, 5, 299.

[8] A. Manthiram , X. Yu , S. Wang , Nat. Rev. Mater. 2017, 2, 16103.

[9] K. Yoon , S. Lee , K. Oh , K. Kang , Adv. Mater. 2022, 34, 2104666. 10.1002/adma.202104666 34747060

[10] Y. Lee , J. Jeong , H. J. Lee , M. Kim , D. Han , H. Kim , J. M. Yuk , K.‐W. Nam , K. Y. Chung , H.‐G. Jung , S. Yu , ACS Energy Lett. 2022, 7, 171.

[11] K. J. Kim , M. Balaish , M. Wadaguchi , L. Kong , J. L. M. Rupp , Adv. Energy Mater. 2021, 11, 2002689.

[12] J. Wu , L. Yuan , Z. Li , X. Xie , Y. Huang , Mater. Horiz. 2020, 7, 2619.

[13] J. Lei , Z. Gao , L. Tang , L. Zhong , J. Li , Y. Zhang , T. Liu , Adv. Sci. 2022, 9, 2103760. 10.1002/advs.202103760 PMC8811808 34894094

[14] D. Geng , N. Ding , T. S. A. Hor , S. W. Chien , Z. Liu , D. Wuu , X. Sun , Y. Zong , Adv. Energy Mater. 2016, 6, 1502164.

[15] J. Christensen , P. Albertus , R. S. Sanchez‐Carrera , T. Lohmann , B. Kozinsky , R. Liedtke , J. Ahmed , A. Kojic , J. The Electrochem. Soc. 2011, 159, R1.

[16] Q. Zhao , S. Stalin , C.‐Z. Zhao , L. A. Archer , Nat. Rev. Mater. 2020, 5, 229.

[17] T. Famprikis , P. Canepa , J. A. Dawson , M. S. Islam , C. Masquelier , Nat. Mater. 2019, 18, 1278. 31427742 10.1038/s41563-019-0431-3

[18] J. Liang , J. Luo , Q. Sun , X. Yang , R. Li , X. Sun , Energy Storage Mater. 2019, 21, 308.

[19] X. Chen , Z. Guan , F. Chu , Z. Xue , F. Wu , Y. Yu , InfoMat 2022, 4, e12248.

[20] R. Chen , Q. Li , X. Yu , L. Chen , H. Li , Chem. Rev. 2020, 120, 6820. 31763824 10.1021/acs.chemrev.9b00268

[21] J. Huang , F. Liang , M. Hou , Y. Zhang , K. Chen , D. Xue , Appl. Mater. Today 2020, 20, 100750.

[22] Q. Liu , Z. Geng , C. Han , Y. Fu , S. Li , Y.‐B. He , F. Kang , B. Li , J. Power Sources 2018, 389, 120.

[23] Y. Nikodimos , C.‐J. Huang , B. W. Taklu , W.‐N. Su , B. J. Hwang , Energy Environ. Sci. 2022, 15, 991.

[24] P. Lu , D. Wu , L. Chen , H. Li , F. Wu , Electrochem. Energy R. 2022, 5, 3.

[25] Y. Nikodimos , W.‐N. Su , B. J. Hwang , Adv. Energy Mater. 2023, 13, 2202854.

[26] U.‐H. Kim , D.‐W. Jun , K.‐J. Park , Q. Zhang , P. Kaghazchi , D. Aurbach , D. T. Major , G. Goobes , M. Dixit , N. Leifer , C. M. Wang , P. Yan , D. Ahn , K.‐H. Kim , C. S. Yoon , Y.‐K. Sun , Energy Environ. Sci. 2018, 11, 1271.

[27] W. He , W. Guo , H. Wu , L. Lin , Q. Liu , X. Han , Q. Xie , P. Liu , H. Zheng , L. Wang , X. Yu , D.‐L. Peng , Adv. Mater. 2021, 33, 2005937. 10.1002/adma.202005937 33772921

[28] J. Liu , J. Wang , Y. Ni , K. Zhang , F. Cheng , J. Chen , Mater. Today 2021, 43, 132.

[29] L. Zou , Y. He , Z. Liu , H. Jia , J. Zhu , J. Zheng , G. Wang , X. Li , J. Xiao , J. Liu , J.i‐G. Zhang , G. Chen , C. Wang , Nat. Commun. 2020, 11, 3204. 32587338 10.1038/s41467-020-17050-6 PMC7316795

[30] Z. Chen , J. Wang , J. Huang , T. Fu , G. Sun , S. Lai , R. Zhou , K. Li , J. Zhao , J. Power Sources 2017, 363, 168.

[31] R. Zhang , S. Yang , H. Li , T. Zhai , H. Li , InfoMat 2022, 4, e12305.

[32] J.u‐M. Kim , X. Zhang , J.i‐G. Zhang , A. Manthiram , Y. S. Meng , W. Xu , Mater. Today 2021, 46, 155.

[33] G.‐L. Xu , Q. Liu , K. K. S. Lau , Y. Liu , X. Liu , H. Gao , X. Zhou , M. Zhuang , Y. Ren , J. Li , M. Shao , M. Ouyang , F. Pan , Z. Chen , K. Amine , G. Chen , Nat. Energy 2019, 4, 484.

[34] J. Lee , S. H. Choi , H. Qutaish , Y. Hyeon , S. A. Han , Y.‐U. Heo , D. Whang , J.‐W. Lee , J. Moon , M.‐S. Park , J. H. Kim , S. X. Dou , Energy Storage Mater. 2021, 37, 315.

[35] K. (.K.). Fu , Y. Gong , B. Liu , Y. Zhu , S. Xu , Y. Yao , W. Luo , C. Wang , S. D. Lacey , J. Dai , Y. Chen , Y. Mo , E. Wachsman , L. Hu , Sci. Adv. 2017, 3, e1601659. 28435874 10.1126/sciadv.1601659 PMC5384807

[36] X. Gao , L. Jiang , Nature 2004, 432, 36. 15525973 10.1038/432036a

[37] T.‐S. Wong , S. H. Kang , S. K. Y. Tang , E. J. Smythe , B. D. Hatton , A. Grinthal , J. Aizenberg , Nature 2011, 477, 443. 21938066 10.1038/nature10447

[38] H. Chen , P. Zhang , L. Zhang , H. Liu , Y. Jiang , D. Zhang , Z. Han , L. Jiang , Nature 2016, 532, 85. 27078568 10.1038/nature17189

[39] M. Yamamoto , N. Nishikawa , H. Mayama , Y. Nonomura , S. Yokojima , S. Nakamura , K. Uchida , Langmuir 2015, 31, 7355. 26075949 10.1021/acs.langmuir.5b00670

[40] J. Wang , H. Chen , T. Sui , A. Li , D. Chen , Plant Sci 2009, 176, 687.

[41] T. Yang , P. Jia , Q. Liu , L. Zhang , C. Du , J. Chen , H. Ye , X. Li , Y. Li , T. Shen , Y. Tang , J. Huang , Angew. Chem., Int. Ed. 2018, 57, 12750. 10.1002/anie.201807355 30063281

[42] J. Wang , H. Hu , S. Duan , Q. Xiao , J. Zhang , H. Liu , Q. Kang , L. Jia , J. Yang , W. Xu , H. Fei , S. Cheng , L. Li , M. Liu , H. Lin , Y. Zhang , Adv. Funct. Mater. 2022, 32, 2110468.

[43] J. Zhang , X. Sheng , L. Jiang , Langmuir 2009, 25, 1371. 19170641 10.1021/la8024233

[44] N. K. Adam , Nature 1957, 180, 809.

[45] D. Zang , R. Zhu , W. Zhang , X. Yu , L. Lin , X. Guo , M. Liu , L. Jiang , Adv. Funct. Mater. 2017, 27, 1605446.

[46] C. Frankiewicz , D. Attinger , Nanoscale 2016, 8, 3982. 26537609 10.1039/c5nr04098a

[47] R. N. Wenzel , Ind. Eng. Chem. 1936, 28, 988.

[48] C. Yang , U. Tartaglino , B. N. J. Persson , Phys. Rev. Lett. 2006, 97, 116103. 17025908 10.1103/PhysRevLett.97.116103

[49] Y. Si , Z. Dong , L. Jiang , ACS Cent. Sci. 2018, 4, 1102. 30276243 10.1021/acscentsci.8b00504 PMC6161061

[50] Y. Zhang , T.‐T. Zuo , J. Popovic , K. Lim , Y.a‐X. Yin , J. Maier , Y.u‐G. Guo , Mater. Today 2020, 33, 56.

[51] J. Liu , Z. Bao , Y. Cui , E. J. Dufek , J. B. Goodenough , P. Khalifah , Q. Li , B. Y. Liaw , P. Liu , A. Manthiram , Y. S. Meng , V. R. Subramanian , M. F. Toney , V. V. Viswanathan , M. S. Whittingham , J. Xiao , W. Xu , J. Yang , X.‐Q. Yang , J.i‐G. Zhang , Nat. Energy 2019, 4, 180.

[52] C. Wang , C. Yang , Z. Zheng , Adv. Sci. 2022, 9, 2105213. 10.1002/advs.202105213 PMC8948585 35098702

[53] A. Hu , F. Li , W. Chen , T. Lei , Y. Li , Y. Fan , M. He , F. Wang , M. Zhou , Y. Hu , Y. Yan , B. Chen , J. Zhu , J. Long , X. Wang , J. Xiong , Adv. Energy Mater. 2022, 12, 2202432.

[54] A. Hu , W. Chen , X. Du , Y. Hu , T. Lei , H. Wang , L. Xue , Y. Li , H. Sun , Y. Yan , J. Long , C. Shu , J. Zhu , B. Li , X. Wang , J. Xiong , Energy Environ. Sci. 2021, 14, 4115.

[55] H. Cheng , Y. Mao , Y. Lu , P. Zhang , J. Xie , X. Zhao , Nanoscale 2020, 12, 3424. 31989997 10.1039/c9nr09749j

[56] Y. Ma , P. Qi , J. Ma , L. Wei , L. Zhao , J. Cheng , Y. Su , Y. Gu , Y. Lian , Y. Peng , Y. Shen , L. Chen , Z. Deng , Z. Liu , Adv. Sci. 2021, 8, 2100488. 10.1002/advs.202100488 PMC8373161 34081418

[57] Q. Xu , J. Lin , C. Ye , X. Jin , D. Ye , Y. Lu , G. Zhou , Y. Qiu , W. Li , Adv. Energy Mater. 2020, 10, 1903292.

[58] X. Shen , Y. Li , T. Qian , J. Liu , J. Zhou , C. Yan , J. B. Goodenough , Nat. Commun. 2019, 10, 900. 30796214 10.1038/s41467-019-08767-0 PMC6385276

[59] T. Chen , F. Meng , Z. Zhang , J. Liang , Y. Hu , W. Kong , X. L. Zhang , Z. Jin , Nano Energy 2020, 76, 105068.

[60] H. Xu , S. Li , X. Chen , C. Zhang , W. Liu , H. Fan , Y. Yu , Y. Huang , J. Li , Adv. Energy Mater. 2019, 9, 1902150.

[61] S. Qu , W. Jia , Y. Wang , C. Li , Z. Yao , K. Li , Y. Liu , W. Zou , F. Zhou , Z. Wang , J. Li , Electrochim. Acta 2019, 317, 120.

[62] K. Lu , H. Xu , H. He , S. Gao , X. Li , C. Zheng , T. Xu , Y. Cheng , J. Mater. Chem. A 2020, 8, 10363.

[63] J. Liang , X. Li , Y. Zhao , L. V. Goncharova , W. Li , K. R. Adair , M. N. Banis , Y. Hu , T.‐K. Sham , H. Huang , L. Zhang , S. Zhao , S. Lu , R. Li , X. Sun , Adv. Energy Mater. 2019, 9, 1902125.

[64] Y. Yu , Y.‐B. Yin , J.‐L. Ma , Z.‐W. Chang , T. Sun , Y.‐H. Zhu , X.‐Y. Yang , T. Liu , X.‐B. Zhang , Energy Storage Mater. 2019, 18, 382.

[65] K. R. Adair , C. Zhao , M. N. Banis , Y. Zhao , R. Li , M. Cai , X. Sun , Angew. Chem., Int. Ed. 2019, 58, 15797. 10.1002/anie.201907759 31400290

[66] Y. Zhang , Y. Liu , J. Zhou , D. Wang , L. Tan , C. Yi , Chem. Eng. J. 2022, 431, 134266.

[67] Y. Zhang , S. Wu , Q.‐H. Yang , EnergyChem 2021, 3, 100063.

[68] Z. Han , C. Zhang , Q. Lin , Y. Zhang , Y. Deng , J. Han , D. Wu , F. Kang , Q.‐H. Yang , W. Lv , Small Methods 2021, 5, 2001035. 10.1002/smtd.202001035 34927844

[69] R.‐M. Gao , H. Yang , C.‐Y. Wang , H. Ye , F.‐F. Cao , Z.‐P. Guo , Angew. Chem., Int. Ed. 2021, 60, 25508. 10.1002/anie.202111199 34580988

[70] Y. Zhu , Y. Zhang , P. Das , Z.‐S. Wu , Energy Fuels 2021, 35, 12902.

[71] Y. Sun , Y. Zhao , J. Wang , J. Liang , C. Wang , Q. Sun , X. Lin , K. R. Adair , J. Luo , D. Wang , R. Li , M. Cai , T.‐K. Sham , X. Sun , Adv. Mater. 2019, 31, 1806541. 10.1002/adma.201806541 30515896

[72] D. Kang , N. Hart , J. Koh , L. Ma , W. Liang , J. Xu , S. Sardar , J. P. Lemmon , Energy Storage Mater. 2020, 24, 618.

[73] S. Li , J. Huang , Y. Cui , S. Liu , Z. Chen , W. Huang , C. Li , R. Liu , R. Fu , D. Wu , Nat. Nanotech. 2022, 17, 613. 10.1038/s41565-022-01107-2 35469010

[74] Y. Han , B. Liu , Z. Xiao , W. Zhang , X. Wang , G. Pan , Y. Xia , X. Xia , J. Tu , InfoMat 2021, 3, 155.

[75] Q. Zhang , S. Liu , Y. Lu , L. Xing , W. Li , J. Energy Chem. 2021, 58, 198.

[76] T. Jesionowski , A. Krysztafkiewicz , Appl. Surf. Sci. 2001, 172, 18.

[77] H. F. Wu , D. W. Dwight , N. T. Huff , Compos. Sci. Technol. 1997, 57, 975.

[78] P. C. Ma , J.‐K. Kim , B. Z. Tang , Carbon 2006, 44, 3232.

[79] Y. Wang , Z. Wang , L. Zhao , Q. Fan , X. Zeng , S. Liu , W. K. Pang , Y.‐B. He , Z. Guo , Adv. Mater. 2021, 33, 2008133. 10.1002/adma.202008133 33656208

[80] T. Kang , Y. Wang , F. Guo , C. Liu , J. Zhao , J. Yang , H. Lin , Y. Qiu , Y. Shen , W. Lu , L. Chen , ACS Cent. Sci. 2019, 5, 468. 30937374 10.1021/acscentsci.8b00845 PMC6439463

[81] Y. Zhang , W. Lv , Z. Huang , G. Zhou , Y. Deng , J. Zhang , C. Zhang , B. Hao , Q. Qi , Y.‐B. He , F. Kang , Q.‐H. Yang , Sci. Bull. 2019, 64, 910. 10.1016/j.scib.2019.05.025 36659755

[82] S. Li , F. Lorandi , J. F. Whitacre , K. Matyjaszewski , Macromol. Chem. Phys. 2020, 221, 1900379.

[83] S.‐J. Zhang , Z.‐G. Gao , W.‐W. Wang , Y.‐Q. Lu , Y.a‐P. Deng , J.‐H. You , J.‐T. Li , Y. Zhou , L. Huang , X.‐D. Zhou , S.‐G. Sun , Small 2018, 14, 1801054.

[84] J. Wu , Z. Rao , X. Liu , Y. Shen , C. Fang , L. Yuan , Z. Li , W. Zhang , X. Xie , Y. Huang , Adv. Mater. 2021, 33, 2007428. 10.1002/adma.202007428 33543568

[85] J. Meng , F. Chu , J. Hu , C. Li , Adv. Funct. Mater. 2019, 29, 1902220.

[86] Q. Yang , W. Li , C. Dong , Y. Ma , Y. Yin , Q. Wu , Z. Xu , W. Ma , C. Fan , K. Sun , J. Energy Chem. 2020, 42, 83.

[87] Y. Zhao , D. Wang , Y. Gao , T. Chen , Q. Huang , D. Wang , Nano Energy 2019, 64, 103893.

[88] Y. Xiao , R. Xu , C. Yan , Y. Liang , J.‐F. Ding , J.‐Q. Huang , Sci. Bull. 2020, 65, 909. 10.1016/j.scib.2020.02.022 36747423

[89] A. Le Mong , D. Kim , J. Power Sources 2021, 495, 229744.

[90] B. Zhao , C. Xing , Y. Shi , Q. Duan , C. Shen , W. Li , Y. Jiang , J. Zhang , J. Colloid Interface Sci. 2023, 642, 193. 37004254 10.1016/j.jcis.2023.03.168

[91] G. Wang , C. Chen , Y. Chen , X. Kang , C. Yang , F. Wang , Y. Liu , X. Xiong , Angew. Chem., Int. Ed. 2020, 59, 2055. 10.1002/anie.201913351 31729145

[92] W. Cao , J. Lu , K. Zhou , G. Sun , J. Zheng , Z. Geng , H. Li , Nano Energy 2022, 95, 106983.

[93] L. Liu , H. Jiang , R. Hu , Z. Shen , H. Li , J. Liu , J. Power Sources 2023, 555, 232395.

[94] G. Li , S. Liu , Z. Liu , Y. Zhao , Small 2021, 17, 2102196. 10.1002/smll.202102196 34323362

[95] Y. Sun , C. Zhao , K. R. Adair , Y. Zhao , L. V. Goncharova , J. Liang , C. Wang , J. Li , R. Li , M. Cai , T.‐K. Sham , X. Sun , Energy Environ. Sci. 2021, 14, 4085.

[96] J. Wang , J. Yang , Q. Xiao , J. Zhang , T. Li , L. Jia , Z. Wang , S. Cheng , L. Li , M. Liu , H. Liu , H. Lin , Y. Zhang , Adv. Funct. Mater. 2021, 31, 2007434.

[97] Z. Jiang , L. Jin , Z. Han , W. Hu , Z. Zeng , Y. Sun , J. Xie , Angew. Chem., Int. Ed. 2019, 58, 11374. 10.1002/anie.201905712 31111996

[98] F. Liu , Q. Xiao , H. B. Wu , L. Shen , D. Xu , M. Cai , Y. Lu , Adv. Energy Mater. 2018, 8, 1701744.

[99] X. Liu , J. Liu , T. Qian , H. Chen , C. Yan , Adv. Mater. 2020, 32, 1902724. 10.1002/adma.201902724 31777980

[100] Y. Liu , R. Hu , D. Zhang , J. Liu , F. Liu , J. Cui , Z. Lin , J. Wu , M. Zhu , Adv. Mater. 2021, 33, 2004711. 10.1002/adma.202004711 33511690

[101] F. Liu , L. Wang , Z. Zhang , P. Shi , Y. Feng , Y. Yao , S. Ye , H. Wang , X. Wu , Y. Yu , Adv. Funct. Mater. 2020, 30, 2001607.

[102] T. Liu , X.i‐L. Feng , X. Jin , M.‐Z. Shao , Y.u‐T. Su , Y. Zhang , X.‐B. Zhang , Angew. Chem., Int. Ed. 2019, 58, 18240. 10.1002/anie.201911229 31588648

[103] K. Zhang , F. Wu , X. Wang , S. Weng , X. Yang , H. Zhao , R. Guo , Y. Sun , W. Zhao , T. Song , X. Wang , Y. Bai , C. Wu , Adv. Energy Mater. 2022, 12, 2200368.

[104] J. Wang , H. Hu , J. Zhang , L. Li , L. Jia , Q. Guan , H. Hu , H. Liu , Y. Jia , Q. Zhuang , S. Cheng , M. Huang , H. Lin , Energy Storage Mater. 2022, 52, 210.

[105] X.‐B. Cheng , R. Zhang , C.‐Z. Zhao , Q. Zhang , Chem. Rev. 2017, 117, 10403. 28753298 10.1021/acs.chemrev.7b00115

[106] H. Ye , Y. Zhang , Y.a‐X. Yin , F.‐F. Cao , Y.u‐G. Guo , ACS Cent. Sci. 2020, 6, 661. 32490184 10.1021/acscentsci.0c00351 PMC7256944

[107] Z.i‐J. Zheng , Q. Su , Q. Zhang , X.‐C. Hu , Y.a‐X. Yin , R. Wen , H. Ye , Z.‐B. Wang , Y.u‐G. Guo , Nano Energy 2019, 64, 103910.

[108] R. Zhang , N.‐W. Li , X.‐B. Cheng , Y.a‐X. Yin , Q. Zhang , Y.u‐G. Guo , Adv. Sci. 2017, 4, 1600445. 10.1002/advs.201600445 PMC5357990 28331792

[109] C.‐Y. Wang , Z.i‐J. Zheng , Y.‐Q. Feng , H. Ye , F.‐F. Cao , Z.‐P. Guo , Nano Energy 2020, 74, 104817.

[110] S.i‐Y. Zeng , C.‐Y. Wang , C. Yang , Z.i‐J. Zheng , ACS Appl. Mater. Interfaces 2022, 14, 41065. 36044205 10.1021/acsami.2c11673

[111] P. Shi , X.‐Q. Zhang , X. Shen , R. Zhang , H. Liu , Q. Zhang , Adv. Mater. Technol. 2020, 5, 1900806.

[112] Y. Huang , J. Duan , X. Zheng , J. Wen , Y. Dai , Z. Wang , W. Luo , Y. Huang , Matter 2020, 3, 1009.

[113] Z.i‐J. Zheng , H. Ye , Z.‐P. Guo , Adv. Sci. 2020, 7, 2002212. 10.1002/advs.202002212 PMC7675197 33240768

[114] J. Zhao , G. Zhou , K. Yan , J. Xie , Y. Li , L. Liao , Y. Jin , K. Liu , P.o‐C. Hsu , J. Wang , H.‐M. Cheng , Y. Cui , Nat. Nanotech. 2017, 12, 993. 10.1038/nnano.2017.129 28692059

[115] L. Dong , L. Nie , W. Liu , Adv. Mater. 2020, 32, 1908494. 10.1002/adma.201908494 32053226

[116] H. Fan , S. Li , Y. Yu , H. Xu , M. Jiang , Y. Huang , J. Li , Adv. Funct. Mater. 2021, 31, 2100978.

[117] S. Li , M. Jiang , Y. Xie , H. Xu , J. Jia , J. Li , Adv. Mater. 2018, 30, 1706375. 10.1002/adma.201706375 29569280

[118] Y. Yu , Y. Liu , J. Xie , ACS Appl. Mater. Interfaces 2021, 13, 18. 33382579 10.1021/acsami.0c17302

[119] Q. Wang , H. Wang , J. Wu , M. Zhou , W. Liu , H. Zhou , Nano Energy 2021, 80, 105516.

[120] Y. Zheng , Y. Yao , J. Ou , M. Li , D. Luo , H. Dou , Z. Li , K. Amine , A. Yu , Z. Chen , Chem. Soc. Rev. 2020, 49, 8790. 33107869 10.1039/d0cs00305k

[121] Z. Gao , H. Sun , L. Fu , F. Ye , Y. Zhang , W. Luo , Y. Huang , Adv. Mater. 2018, 30, 1705702. 10.1002/adma.201705702 29468745

[122] W. Zhao , J. Yi , P. He , H. Zhou , Electrochem. Energy R. 2019, 2, 574.

[123] C. Wang , K. Fu , S. P. Kammampata , D. W. Mcowen , A. J. Samson , L. Zhang , G. T. Hitz , A. M. Nolan , E. D. Wachsman , Y. Mo , V. Thangadurai , L. Hu , Chem. Rev. 2020, 120, 4257. 32271022 10.1021/acs.chemrev.9b00427

[124] Q. Zhang , D. Cao , Y. Ma , A. Natan , P. Aurora , H. Zhu , Adv. Mater. 2019, 31, 1901131. 10.1002/adma.201901131 31441140

[125] J. Lee , T. Lee , K. Char , K. J. Kim , J. W. Choi , Acc. Chem. Res. 2021, 54, 3390. 34402619 10.1021/acs.accounts.1c00333

[126] J. Zhao , X. Wang , T. Wei , Z. Zhang , G. Liu , W. Yu , X. Dong , J. Wang , J. Energy Storage 2023, 68, 107693.

[127] X. Miao , H. Wang , R. Sun , C. Wang , Z. Zhang , Z. Li , L. Yin , Energy Environ. Sci. 2020, 13, 3780.

[128] J. Dai , C. Yang , C. Wang , G. Pastel , L. Hu , Adv. Mater. 2018, 30, 1802068. 10.1002/adma.201802068 30302834

[129] V. Siller , A. Morata , M. N. Eroles , R. Arenal , J. C. Gonzalez‐Rosillo , J. M. López Del Amo , A. Tarancón , J. Mater. Chem. A 2021, 9, 17760.

[130] S. Abouali , C.‐H. Yim , A. Merati , Y. Abu‐Lebdeh , V. Thangadurai , ACS Energy Lett. 2021, 6, 1920.

[131] C. Galven , J.‐L. Fourquet , M.‐P. Crosnier‐Lopez , F. Le Berre , Chem. Mater. 2011, 23, 1892.

[132] W. Guo , F. Shen , J. Liu , Q. Zhang , H. Guo , Y. Yin , J. Gao , Z. Sun , X. Han , Y. Hu , Energy Storage Mater. 2021, 41, 791.

[133] L. H. Abrha , T. T. Hagos , Y. Nikodimos , H. K. Bezabh , G. B. Berhe , T. M. Hagos , C.‐J. Huang , W. A. Tegegne , S.‐K. Jiang , H. H. Weldeyohannes , S.‐H. Wu , W.‐N. Su , B. J. Hwang , ACS Appl. Mater. Interfaces 2020, 12, 25709. 32407073 10.1021/acsami.0c01289

[134] Amardeep , S. Kobi , A. Mukhopadhyay , Scripta Mater 2019, 162, 214.

[135] D. Wang , C. Zhu , Y. Fu , X. Sun , Y. Yang , Adv. Energy Mater. 2020, 10, 2001318.

[136] T. Wang , W. Luo , Y. Huang , Acc. Chem. Res. 2023, 56, 667. 36848173 10.1021/acs.accounts.2c00822

[137] S. Rajendran , A. Pilli , O. Omolere , J. Kelber , L. M. R. Arava , Chem. Mater. 2021, 33, 3401.

[138] H. Duan , W.‐P. Chen , M. Fan , W.‐P. Wang , L. Yu , S.‐J. Tan , X. Chen , Q. Zhang , S. Xin , L.i‐J. Wan , Y.u‐G. Guo , Angew. Chem., Int. Ed. 2020, 59, 12069. 10.1002/anie.202003177 32294296

[139] W. Feng , X. Dong , X. Zhang , Z. Lai , P. Li , C. Wang , Y. Wang , Y. Xia , Angew. Chem., Int. Ed. 2020, 59, 5346. 10.1002/anie.201915900 31965702

[140] Z. Bi , Q. Sun , M. Jia , M. Zuo , N. Zhao , X. Guo , Adv. Funct. Mater. 2022, 32, 2208751.

[141] M. Khurram Tufail , N. Ahmad , L. Zhou , M. Faheem , L. Yang , R. Chen , W. Yang , Chem. Eng. J. 2021, 425, 130535.

[142] D. Liu , W. Zhu , Z. Feng , A. Guerfi , A. Vijh , K. Zaghib , Mat. Sci. Eng. B 2016, 213, 169.

[143] H. Liu , Y. Liang , C. Wang , D. Li , X. Yan , C.e‐W. Nan , L.i‐Z. Fan , Adv. Mater. 2023, 2206013. 10.1002/adma.202206013 35984755

[144] R. G. Pearson , J. Am. Chem. Soc. 1963, 85, 3533.

[145] F. Zhao , J. Liang , C. Yu , Q. Sun , X. Li , K. Adair , C. Wang , Y. Zhao , S. Zhang , W. Li , S. Deng , R. Li , Y. Huang , H. Huang , L. Zhang , S. Zhao , S. Lu , X. Sun , Adv. Energy Mater. 2020, 10, 1903422.

[146] F. Zhao , S. H. Alahakoon , K. Adair , S. Zhang , W. Xia , W. Li , C. Yu , R. Feng , Y. Hu , J. Liang , X. Lin , Y. Zhao , X. Yang , T.‐K. Sham , H. Huang , L. Zhang , S. Zhao , S. Lu , Y. Huang , X. Sun , Adv. Mater. 2021, 33, 2006577. 10.1002/adma.202006577 33470466

[147] J. Liang , N. Chen , X. Li , X. Li , K. R. Adair , J. Li , C. Wang , C. Yu , M. Norouzi Banis , L. Zhang , S. Zhao , S. Lu , H. Huang , R. Li , Y. Huang , X. Sun , Chem. Mater. 2020, 32, 2664.

[148] M. K. Tufail , L. Zhou , N. Ahmad , R. Chen , M. Faheem , L. Yang , W. Yang , Chem. Eng. J. 2021, 407, 127149.

[149] L. Ye , E. Gil‐González , X. Li , Electrochem. Commun. 2021, 128, 107058.

[150] G. Liu , D. Xie , X. Wang , X. Yao , S. Chen , R. Xiao , H. Li , X. Xu , Energy Storage Mater. 2019, 17, 266.

[151] N. Ahmad , L. Zhou , M. Faheem , M. K. Tufail , L. Yang , R. Chen , Y. Zhou , W. Yang , ACS Appl. Mater. Interfaces 2020, 12, 21548. 32286785 10.1021/acsami.0c00393

[152] K. H. Park , Q. Bai , D. H. Kim , D. Y. Oh , Y. Zhu , Y. Mo , Y. S. Jung , Adv. Energy Mater. 2018, 8, 1800035.

[153] J. Xu , Y. Li , P. Lu , W. Yan , M. Yang , H. Li , L. Chen , F. Wu , Adv. Energy Mater. 2022, 12, 2102348.

[154] Y. Jin , Q. He , G. Liu , Z. Gu , M. Wu , T. Sun , Z. Zhang , L. Huang , X. Yao , Adv. Mater. 2023, 35, 2211047. 10.1002/adma.202211047 36906926

[155] N. Ahmad , S. Sun , P. Yu , W. Yang , Adv. Funct. Mater. 2022, 32, 2201528.

[156] X. Li , J. Liang , J. Luo , M. Norouzi Banis , C. Wang , W. Li , S. Deng , C. Yu , F. Zhao , Y. Hu , T.‐K. Sham , L. Zhang , S. Zhao , S. Lu , H. Huang , R. Li , K. R. Adair , X. Sun , Energy Environ. Sci. 2019, 12, 2665.

[157] X. Chen , Z. Jia , H. Lv , C. Wang , N. Zhao , X. Guo , J. Power Sources 2022, 545, 231939.

[158] B. Tao , D. Zhong , H. Li , G. Wang , H. Chang , Chem. Sci. 2023, 14, 8693. 37621443 10.1039/d3sc02093b PMC10445474

[159] X. Luo , X. Hu , Y. Zhong , X. Wang , J. Tu , Small 2023, n/a, 2306736.

[160] W. Li , J. Liang , M. Li , K. R. Adair , X. Li , Y. Hu , Q. Xiao , R. Feng , R. Li , L. Zhang , S. Lu , H. Huang , S. Zhao , T.‐K. Sham , X. Sun , Chem. Mater. 2020, 32, 7019.

[161] S. Wang , X. Xu , C. Cui , C. Zeng , J. Liang , J. Fu , R. Zhang , T. Zhai , H. Li , Adv. Funct. Mater. 2022, 32, 2108805.

[162] G. H. Chun , J. H. Shim , S. Yu , ACS Appl. Mater. Interfaces 2022, 14, 1241. 34951299 10.1021/acsami.1c22104

[163] M. S. Whittingham , Chem. Rev. 2004, 104, 4271. 15669156 10.1021/cr020731c

[164] G.‐L. Xu , X. Liu , A. Daali , R. Amine , Z. Chen , K. Amine , Adv. Funct. Mater. 2020, 30, 2004748.

[165] M. R. Kaiser , Z. Han , J. Liang , S.‐X. Dou , J. Wang , Energy Storage Mater. 2019, 19, 1.

[166] X. Zheng , Z. Cai , J. Sun , J. He , W. Rao , J. Wang , Y. Zhang , Q. Gao , B. Han , K. Xia , R. Sun , C. Zhou , J. Energy Storage 2023, 58, 106405.

[167] A. Manthiram , B. Song , W. Li , Energy Storage Mater. 2017, 6, 125.

[168] Y. Zhu , Y. Mo , Angew. Chem., Int. Ed. 2020, 59, 17472. 10.1002/anie.202007621 32597549

[169] F. Zhang , Q. Han , D. Xiong , S. Wang , J. Solid State Electrochem. 2023, 27, 2227.

[170] Y. You , H. Celio , J. Li , A. Dolocan , A. Manthiram , Angew. Chem., Int. Ed. 2018, 57, 6480. 10.1002/anie.201801533 29601125

[171] P. Hou , J. Yin , M. Ding , J. Huang , X. Xu , Small 2017, 13, 1701802. 10.1002/smll.201701802 28977732

[172] H. Sheng , X.‐H. Meng , D.‐D. Xiao , M. Fan , W.‐P. Chen , J. Wan , J. Tang , Y.u‐G. Zou , F. Wang , R. Wen , J.i‐L. Shi , Y.u‐G. Guo , Adv. Mater. 2022, 34, 2108947. 10.1002/adma.202108947 34994990

[173] Y. Huang , P.‐Y. Li , H.‐X. Wei , Y.u‐H. Luo , L.‐B. Tang , H.e‐Z. Chen , X.‐H. Zhang , J.‐C. Zheng , Chem. Eng. J. 2023, 477, 146850.

[174] Y. Mu , H. Ming , X. Chen , W. Zhang , G. Cao , Y. Xiang , J. Qiu , Adv. Sustain. Syst. 2022, 6, 2200002.

[175] S. Li , D. Leng , W. Li , L. Qie , Z. Dong , Z. Cheng , Z. Fan , Energy Storage Mater. 2020, 27, 279.

[176] D. Su , D. Zhou , C. Wang , G. Wang , Adv. Funct. Mater. 2018, 28, 1800154.

[177] J. Xiang , Y. Zhao , L. Wang , C. Zha , J. Mater. Chem. A 2022, 10, 10326.

[178] J. Jiang , Q. Fan , S. Chou , Z. Guo , K. Konstantinov , H. Liu , J. Wang , Small 2021, 17, 1903934. 10.1002/smll.201903934 31657137

[179] V. Brune , C. Bohr , T. Ludwig , M. Wilhelm , S. D. Hirt , T. Fischer , S. Wennig , B. Oberschachtsiek , A. Ichangi , S. Mathur , J. Mater. Chem. A 2022, 10, 9902.

[180] J. Zhang , E. Xu , Z. Sun , Y. Zhou , P. Shi , Y. Gao , Z. Bao , Y. Jiang , Small Methods 2020, 4, 2000463.

[181] M. Yu , S. Zhou , Z. Wang , W. Pei , X. Liu , C. Liu , C. Yan , X. Meng , S. Wang , J. Zhao , J. Qiu , Adv. Funct. Mater. 2019, 29, 1905986.

[182] X. Meng , Y. Liu , L. Yu , J. Qiu , Z. Wang , Adv. Funct. Mater. 2023, 33, 2211062.

[183] C. Li , N. Li , X. Zhang , Z. Dong , H. Chen , L. Jiang , Angew. Chem., Int. Ed. 2016, 55, 14988. 10.1002/anie.201607514 27654652

[184] P. Zhang , L. Zhang , H. Chen , Z. Dong , D. Zhang , Adv. Mater. 2017, 29, 1702995. 10.1002/adma.201702995 28782892

